# 6-Methyl-2-*p*-tolyl-4-[3-(trifluoro­meth­yl)phen­yl]pyridazin-3(2*H*)-one

**DOI:** 10.1107/S1600536808005928

**Published:** 2008-03-07

**Authors:** Zi-Xia Niu, You-Quan Zhu, Fang-Zhong Hu, Xu-Hong Hu, Hua-Zheng Yang

**Affiliations:** a State Key Laboratory and Institute of Elemento-Organic Chemistry, Nankai University, Tianjin 300071, People’s Republic of China

## Abstract

In the title mol­ecule, C_19_H_15_F_3_N_2_O, the benzene rings of the tolyl and trifluoro­methyl­phenyl groups form dihedral angles of 64.1 (2) and 38.5 (2)°, respectively, with the pyridazine ring. The CF_3_ group is disordered over two orientations, with site-occupancy factors of *ca* 0.56 and 0.44.

## Related literature

For related literature, see: Heinisch & Kopelent (1992 ▶); Kolar & Tisler (1999 ▶).
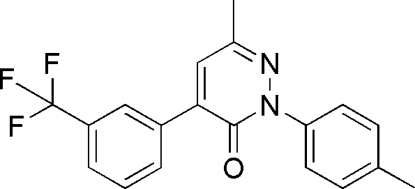


## Experimental

### 

#### Crystal data

C_19_H_15_F_3_N_2_O
*M* *_r_* = 344.33
Monoclinic, 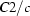

*a* = 20.902 (6) Å
*b* = 4.2898 (13) Å
*c* = 37.683 (11) Å
β = 101.534 (5)°
*V* = 3310.6 (17) Å^3^
*Z* = 8
Mo *K*α radiation
μ = 0.11 mm^−1^
*T* = 294 (2) K
0.52 × 0.20 × 0.16 mm

#### Data collection

Bruker SMART CCD area-detector diffractometer
Absorption correction: multi-scan (*SADABS*; Sheldrick, 1996 ▶) *T*_min_ = 0.945, *T*_max_ = 0.983
7702 measured reflections
2907 independent reflections
1988 reflections with *I* > 2σ(*I*)
*R*_int_ = 0.035

#### Refinement

*R*[*F* ^2^ > 2σ(*F* ^2^)] = 0.059
*wR*(*F* ^2^) = 0.170
*S* = 1.05
2907 reflections
256 parameters
51 restraints
H-atom parameters constrained
Δρ_max_ = 0.20 e Å^−3^
Δρ_min_ = −0.25 e Å^−3^

### 

Data collection: *SMART* (Bruker, 1999 ▶); cell refinement: *SAINT* (Bruker, 1999 ▶); data reduction: *SAINT*; program(s) used to solve structure: *SHELXS97* (Sheldrick, 2008 ▶); program(s) used to refine structure: *SHELXL97* (Sheldrick, 2008 ▶); molecular graphics: *SHELXTL* (Sheldrick, 2008 ▶); software used to prepare material for publication: *SHELXL97*.

## Supplementary Material

Crystal structure: contains datablocks global, I. DOI: 10.1107/S1600536808005928/ci2564sup1.cif

Structure factors: contains datablocks I. DOI: 10.1107/S1600536808005928/ci2564Isup2.hkl

Additional supplementary materials:  crystallographic information; 3D view; checkCIF report

## Figures and Tables

**Fig. 1.**
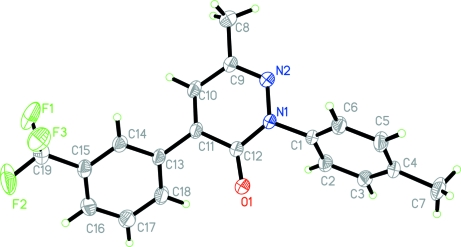
The molecular structure of the title compound, showing 30% probability displacement ellipsoids. Only one disorder component is shown.

## supplementary crystallographic information

### Comment

Many pyridazine derivatives have been found to exhibit biological activities such as insecticidal, fungicidal, herbicidal, plant-growth regulatory activity, *etc*. (Heinisch & Kopelent, 1992). For example, pyridate, credazine and maleic hydrazide (Kolar & Tisler, 1999) have been commercialized as herbicides. In order to discover new biologically active pyridazine compounds, the title compound was synthesized and its structure is reported here.

In the molecule of the title compound (Fig. 1), the central pyridazine ring (C9—C12/N1/N2) forms dihedral angles of 64.1 (2)° and 38.5 (2)°, respectively, with the C1—C6 and C13—C18 benzene rings. The C2—C1—N1—N2, C2—C1—N1—C12, C6—C1—N1—N2, C6—C1—N1—C12, C10—C11—C13—C14, C12—C11—C13—C14, C10—C11—C13—C18 and C12—C11—C13—C18 torsion angles are -115.7 (3), 65.2 (4), 64.5 (4), -114.6 (3), -38.5 (5), 140.2 (3), 141.6 (3) and -39.7 (5)°, respectively. No significant hydrogen bonding interactions are observed in the crystal structure.

### Experimental

A mixture of ethyl 2-(3-trifluoromethylphenyl)-4-oxopentanoate (2.3 mmol) and 4-methylphenylhydrazine (2.3 mmol) and glacial acetic acid (1 ml) was stirred at room temperature for 2 h. The precipitate formed was filtered and recrystallized from ethanol. Single crystals suitable for X-ray analysis were grown from a ethyl acetate-petroleum ether (3:1 *v*/*v*) solution at room temperature.

### Refinement

The trifluoromethyl group is disordered over two orienatations (C19/F1/F2/F3 and C19/F1'/F2'/F3') with refined occupancies of 0.564 (15) and 0.436 (15). All C—F distances were restrained to be equal and the U^ij^ components of disordered F atoms were restrained to be approximately isotropic. The H atoms were positioned geometrically (C—H = 0.93 or 0.96 Å) and included in the final cycles of refinement using a riding model, with *U*_iso_(H) = 1.2*U*_eq_(C) or 1.5*U*_eq_(methyl C).

### Crystal data

**Table:**

C_19_H_15_F_3_N_2_O	*F*_000_ = 1424
*M**_r_* = 344.33	*D*_x_ = 1.382 Mg m^−^^3^
Monoclinic, *C*2/*c*	Mo *K*α radiation λ = 0.71073 Å
Hall symbol: -C 2yc	Cell parameters from 2694 reflections
*a* = 20.902 (6) Å	θ = 2.2–26.2º
*b* = 4.2898 (13) Å	µ = 0.11 mm^−^^1^
*c* = 37.683 (11) Å	*T* = 294 (2) K
β = 101.534 (5)º	Block, colourless
*V* = 3310.6 (17) Å^3^	0.52 × 0.20 × 0.16 mm
*Z* = 8	

### Data collection

**Table:**

Bruker SMART CCD area-detector diffractometer	2907 independent reflections
Radiation source: fine-focus sealed tube	1988 reflections with *I* > 2σ(*I*)
Monochromator: graphite	*R*_int_ = 0.035
*T* = 294(2) K	θ_max_ = 25.0º
φ and ω scans	θ_min_ = 1.1º
Absorption correction: multi-scan(SADABS; Sheldrick, 1996)	*h* = −24→17
*T*_min_ = 0.945, *T*_max_ = 0.983	*k* = −5→4
7702 measured reflections	*l* = −36→44

### Refinement

**Table:**

Refinement on *F*^2^	Secondary atom site location: difference Fourier map
Least-squares matrix: full	Hydrogen site location: inferred from neighbouring sites
*R*[*F*^2^ > 2σ(*F*^2^)] = 0.059	H-atom parameters constrained
*wR*(*F*^2^) = 0.170	*w* = 1/[σ^2^(*F*_o_^2^) + (0.0755*P*)^2^ + 3.8729*P*] where *P* = (*F*_o_^2^ + 2*F*_c_^2^)/3
*S* = 1.05	(Δ/σ)_max_ = 0.002
2907 reflections	Δρ_max_ = 0.20 e Å^−^^3^
256 parameters	Δρ_min_ = −0.25 e Å^−^^3^
51 restraints	Extinction correction: none
Primary atom site location: structure-invariant direct methods	

### Special details

**Table:**

Geometry. All e.s.d.'s (except the e.s.d. in the dihedral angle between two l.s. planes) are estimated using the full covariance matrix. The cell e.s.d.'s are taken into account individually in the estimation of e.s.d.'s in distances, angles and torsion angles; correlations between e.s.d.'s in cell parameters are only used when they are defined by crystal symmetry. An approximate (isotropic) treatment of cell e.s.d.'s is used for estimating e.s.d.'s involving l.s. planes.
Refinement. Refinement of *F*^2^ against ALL reflections. The weighted *R*-factor *wR* and goodness of fit *S* are based on *F*^2^, conventional *R*-factors *R* are based on *F*, with *F* set to zero for negative *F*^2^. The threshold expression of *F*^2^ > σ(*F*^2^) is used only for calculating *R*-factors(gt) *etc*. and is not relevant to the choice of reflections for refinement. *R*-factors based on *F*^2^ are statistically about twice as large as those based on *F*, and *R*- factors based on ALL data will be even larger.

### Fractional atomic coordinates and isotropic or equivalent isotropic displacement parameters (Å^2^)

**Table:**

	*x*	*y*	*z*	*U*_iso_*/*U*_eq_	Occ. (<1)
O1	0.15529 (10)	0.4998 (7)	0.16676 (6)	0.0680 (8)	
N1	0.22749 (11)	0.2163 (6)	0.14191 (6)	0.0444 (6)	
N2	0.24743 (11)	0.0589 (6)	0.11419 (7)	0.0474 (7)	
C1	0.27804 (13)	0.2541 (8)	0.17437 (8)	0.0423 (7)	
C2	0.27033 (14)	0.1120 (9)	0.20615 (8)	0.0529 (9)	
H2	0.2336	−0.0092	0.2067	0.064*	
C3	0.31796 (14)	0.1520 (9)	0.23731 (8)	0.0530 (9)	
H3	0.3127	0.0567	0.2587	0.064*	
C4	0.37291 (14)	0.3305 (8)	0.23708 (8)	0.0498 (8)	
C5	0.37977 (15)	0.4657 (9)	0.20447 (9)	0.0583 (9)	
H5	0.4170	0.5823	0.2037	0.070*	
C6	0.33258 (14)	0.4312 (8)	0.17308 (8)	0.0514 (8)	
H6	0.3377	0.5256	0.1516	0.062*	
C7	0.42303 (18)	0.3813 (12)	0.27183 (9)	0.0780 (12)	
H7A	0.4027	0.3520	0.2923	0.117*	
H7B	0.4400	0.5894	0.2721	0.117*	
H7C	0.4581	0.2344	0.2730	0.117*	
C8	0.22624 (17)	−0.1579 (10)	0.05424 (9)	0.0675 (10)	
H8A	0.2697	−0.2353	0.0627	0.101*	
H8B	0.2261	−0.0166	0.0345	0.101*	
H8C	0.1974	−0.3291	0.0462	0.101*	
C9	0.20357 (14)	0.0106 (8)	0.08460 (8)	0.0471 (8)	
C10	0.13750 (14)	0.1112 (8)	0.08120 (8)	0.0475 (8)	
H10	0.1075	0.0670	0.0600	0.057*	
C11	0.11739 (13)	0.2704 (8)	0.10827 (8)	0.0432 (7)	
C12	0.16574 (13)	0.3414 (8)	0.14129 (8)	0.0469 (8)	
C13	0.04906 (13)	0.3800 (8)	0.10537 (8)	0.0445 (8)	
C14	0.01367 (14)	0.4972 (7)	0.07268 (8)	0.0463 (8)	
H14	0.0333	0.5083	0.0526	0.056*	
C15	−0.05036 (14)	0.5971 (8)	0.06965 (9)	0.0488 (8)	
C16	−0.08032 (16)	0.5813 (9)	0.09917 (10)	0.0613 (10)	
H16	−0.1233	0.6468	0.0972	0.074*	
C17	−0.04563 (16)	0.4667 (11)	0.13184 (10)	0.0695 (11)	
H17	−0.0656	0.4552	0.1517	0.083*	
C18	0.01841 (15)	0.3695 (10)	0.13515 (9)	0.0599 (10)	
H18	0.0413	0.2966	0.1573	0.072*	
C19	−0.08649 (16)	0.7180 (8)	0.03406 (11)	0.0651 (10)	
F1	−0.0909 (6)	0.5095 (13)	0.00586 (14)	0.100 (3)	0.564 (15)
F2	−0.1439 (3)	0.841 (3)	0.0327 (3)	0.119 (4)	0.564 (15)
F3	−0.0530 (5)	0.943 (2)	0.0207 (3)	0.083 (3)	0.564 (15)
F1'	−0.1325 (6)	0.5147 (17)	0.0190 (3)	0.100 (4)	0.436 (15)
F2'	−0.1258 (5)	0.955 (2)	0.0415 (3)	0.080 (3)	0.436 (15)
F3'	−0.0510 (6)	0.834 (3)	0.0126 (3)	0.095 (4)	0.436 (15)

### Atomic displacement parameters (Å^2^)

**Table:**

	*U*^11^	*U*^22^	*U*^33^	*U*^12^	*U*^13^	*U*^23^
O1	0.0453 (12)	0.105 (2)	0.0480 (14)	0.0123 (13)	−0.0030 (10)	−0.0287 (14)
N1	0.0358 (12)	0.0615 (17)	0.0327 (13)	0.0009 (12)	−0.0004 (10)	−0.0072 (12)
N2	0.0439 (14)	0.0591 (17)	0.0378 (14)	0.0016 (12)	0.0047 (11)	−0.0052 (12)
C1	0.0339 (14)	0.056 (2)	0.0336 (16)	0.0044 (14)	−0.0004 (12)	−0.0054 (14)
C2	0.0406 (16)	0.071 (2)	0.0455 (19)	−0.0073 (16)	0.0049 (14)	0.0000 (17)
C3	0.0484 (18)	0.072 (2)	0.0375 (17)	0.0012 (17)	0.0049 (14)	0.0023 (16)
C4	0.0384 (16)	0.067 (2)	0.0390 (18)	0.0033 (15)	−0.0039 (13)	−0.0042 (16)
C5	0.0400 (16)	0.081 (3)	0.050 (2)	−0.0136 (17)	−0.0008 (15)	0.0027 (18)
C6	0.0443 (17)	0.067 (2)	0.0405 (17)	−0.0022 (16)	0.0029 (14)	0.0062 (16)
C7	0.061 (2)	0.117 (4)	0.046 (2)	−0.017 (2)	−0.0133 (17)	0.003 (2)
C8	0.066 (2)	0.085 (3)	0.050 (2)	0.006 (2)	0.0058 (17)	−0.019 (2)
C9	0.0447 (17)	0.056 (2)	0.0370 (16)	−0.0016 (15)	0.0000 (13)	−0.0030 (15)
C10	0.0441 (16)	0.057 (2)	0.0360 (17)	−0.0083 (15)	−0.0044 (13)	−0.0038 (15)
C11	0.0370 (15)	0.0545 (19)	0.0344 (16)	−0.0029 (14)	−0.0021 (12)	0.0016 (14)
C12	0.0371 (15)	0.064 (2)	0.0374 (17)	0.0014 (15)	0.0011 (13)	−0.0063 (16)
C13	0.0344 (15)	0.058 (2)	0.0372 (16)	−0.0064 (14)	−0.0019 (13)	−0.0015 (15)
C14	0.0399 (16)	0.0546 (19)	0.0419 (17)	−0.0078 (14)	0.0021 (13)	−0.0041 (15)
C15	0.0392 (16)	0.052 (2)	0.0497 (19)	−0.0034 (14)	−0.0043 (14)	−0.0023 (15)
C16	0.0383 (16)	0.079 (3)	0.065 (2)	0.0013 (17)	0.0052 (16)	−0.009 (2)
C17	0.0475 (19)	0.107 (3)	0.055 (2)	−0.001 (2)	0.0135 (16)	0.000 (2)
C18	0.0441 (17)	0.090 (3)	0.0422 (18)	−0.0023 (18)	0.0011 (14)	0.0086 (19)
C19	0.059 (2)	0.055 (2)	0.071 (3)	0.0049 (19)	−0.010 (2)	0.002 (2)
F1	0.141 (7)	0.080 (3)	0.056 (3)	−0.001 (4)	−0.034 (3)	0.005 (2)
F2	0.044 (3)	0.190 (9)	0.117 (6)	0.025 (4)	0.004 (3)	0.065 (6)
F3	0.098 (5)	0.083 (4)	0.065 (5)	−0.008 (4)	0.012 (3)	0.011 (3)
F1'	0.098 (6)	0.079 (4)	0.091 (6)	−0.012 (4)	−0.055 (5)	−0.008 (4)
F2'	0.066 (5)	0.069 (4)	0.094 (5)	0.021 (4)	−0.011 (4)	0.005 (3)
F3'	0.087 (5)	0.155 (9)	0.044 (5)	0.050 (6)	0.016 (4)	0.024 (6)

### Geometric parameters (Å, °)

**Table:**

O1—C12	1.231 (4)	C9—C10	1.428 (4)
N1—N2	1.377 (3)	C10—C11	1.362 (4)
N1—C12	1.394 (4)	C10—H10	0.93
N1—C1	1.457 (3)	C11—C12	1.470 (4)
N2—C9	1.311 (4)	C11—C13	1.487 (4)
C1—C6	1.379 (4)	C13—C14	1.398 (4)
C1—C2	1.382 (4)	C13—C18	1.400 (4)
C2—C3	1.390 (4)	C14—C15	1.388 (4)
C2—H2	0.93	C14—H14	0.93
C3—C4	1.382 (5)	C15—C16	1.383 (5)
C3—H3	0.93	C15—C19	1.494 (5)
C4—C5	1.392 (5)	C16—C17	1.388 (5)
C4—C7	1.520 (4)	C16—H16	0.93
C5—C6	1.388 (4)	C17—C18	1.384 (5)
C5—H5	0.93	C17—H17	0.93
C6—H6	0.93	C18—H18	0.93
C7—H7A	0.96	C19—F3'	1.300 (7)
C7—H7B	0.96	C19—F2	1.303 (6)
C7—H7C	0.96	C19—F1'	1.337 (6)
C8—C9	1.508 (4)	C19—F3	1.347 (6)
C8—H8A	0.96	C19—F2'	1.370 (6)
C8—H8B	0.96	C19—F1	1.377 (6)
C8—H8C	0.96		
			
N2—N1—C12	126.6 (2)	O1—C12—N1	120.5 (3)
N2—N1—C1	114.4 (2)	O1—C12—C11	125.1 (3)
C12—N1—C1	119.0 (2)	N1—C12—C11	114.3 (3)
C9—N2—N1	117.2 (2)	C14—C13—C18	118.2 (3)
C6—C1—C2	120.9 (3)	C14—C13—C11	120.7 (3)
C6—C1—N1	119.9 (3)	C18—C13—C11	121.2 (3)
C2—C1—N1	119.2 (3)	C15—C14—C13	121.0 (3)
C1—C2—C3	119.3 (3)	C15—C14—H14	119.5
C1—C2—H2	120.3	C13—C14—H14	119.5
C3—C2—H2	120.3	C16—C15—C14	120.2 (3)
C4—C3—C2	121.3 (3)	C16—C15—C19	120.7 (3)
C4—C3—H3	119.4	C14—C15—C19	119.1 (3)
C2—C3—H3	119.4	C15—C16—C17	119.4 (3)
C3—C4—C5	118.0 (3)	C15—C16—H16	120.3
C3—C4—C7	120.3 (3)	C17—C16—H16	120.3
C5—C4—C7	121.7 (3)	C18—C17—C16	120.7 (3)
C6—C5—C4	121.8 (3)	C18—C17—H17	119.6
C6—C5—H5	119.1	C16—C17—H17	119.6
C4—C5—H5	119.1	C17—C18—C13	120.5 (3)
C1—C6—C5	118.7 (3)	C17—C18—H18	119.7
C1—C6—H6	120.6	C13—C18—H18	119.7
C5—C6—H6	120.6	F3'—C19—F2	117.1 (7)
C4—C7—H7A	109.5	F3'—C19—F1'	115.9 (8)
C4—C7—H7B	109.5	F2—C19—F1'	70.8 (5)
H7A—C7—H7B	109.5	F3'—C19—F3	24.5 (7)
C4—C7—H7C	109.5	F2—C19—F3	103.7 (7)
H7A—C7—H7C	109.5	F1'—C19—F3	133.8 (7)
H7B—C7—H7C	109.5	F3'—C19—F2'	106.5 (8)
C9—C8—H8A	109.5	F2—C19—F2'	28.5 (5)
C9—C8—H8B	109.5	F1'—C19—F2'	99.3 (6)
H8A—C8—H8B	109.5	F3—C19—F2'	85.6 (6)
C9—C8—H8C	109.5	F3'—C19—F1	74.2 (6)
H8A—C8—H8C	109.5	F2—C19—F1	108.3 (5)
H8B—C8—H8C	109.5	F1'—C19—F1	47.0 (4)
N2—C9—C10	121.9 (3)	F3—C19—F1	97.8 (5)
N2—C9—C8	116.7 (3)	F2'—C19—F1	133.5 (5)
C10—C9—C8	121.4 (3)	F3'—C19—C15	116.2 (6)
C11—C10—C9	121.5 (3)	F2—C19—C15	118.2 (5)
C11—C10—H10	119.3	F1'—C19—C15	110.2 (5)
C9—C10—H10	119.3	F3—C19—C15	112.1 (5)
C10—C11—C12	118.4 (3)	F2'—C19—C15	106.8 (5)
C10—C11—C13	122.6 (3)	F1—C19—C15	114.3 (3)
C12—C11—C13	119.0 (3)		
			
C12—N1—N2—C9	−2.8 (5)	C10—C11—C12—N1	−4.0 (4)
C1—N1—N2—C9	178.3 (3)	C13—C11—C12—N1	177.3 (3)
N2—N1—C1—C6	64.5 (4)	C10—C11—C13—C14	−38.5 (5)
C12—N1—C1—C6	−114.6 (3)	C12—C11—C13—C14	140.2 (3)
N2—N1—C1—C2	−115.7 (3)	C10—C11—C13—C18	141.6 (3)
C12—N1—C1—C2	65.2 (4)	C12—C11—C13—C18	−39.7 (5)
C6—C1—C2—C3	0.8 (5)	C18—C13—C14—C15	−0.7 (5)
N1—C1—C2—C3	−178.9 (3)	C11—C13—C14—C15	179.3 (3)
C1—C2—C3—C4	−0.1 (5)	C13—C14—C15—C16	−0.2 (5)
C2—C3—C4—C5	−1.0 (5)	C13—C14—C15—C19	−179.5 (3)
C2—C3—C4—C7	177.6 (3)	C14—C15—C16—C17	0.4 (5)
C3—C4—C5—C6	1.6 (5)	C19—C15—C16—C17	179.7 (3)
C7—C4—C5—C6	−177.0 (4)	C15—C16—C17—C18	0.2 (6)
C2—C1—C6—C5	−0.3 (5)	C16—C17—C18—C13	−1.1 (6)
N1—C1—C6—C5	179.5 (3)	C14—C13—C18—C17	1.3 (5)
C4—C5—C6—C1	−0.9 (5)	C11—C13—C18—C17	−178.7 (3)
N1—N2—C9—C10	−1.1 (5)	C16—C15—C19—F3'	155.1 (8)
N1—N2—C9—C8	179.1 (3)	C14—C15—C19—F3'	−25.6 (9)
N2—C9—C10—C11	1.9 (5)	C16—C15—C19—F2	7.9 (9)
C8—C9—C10—C11	−178.3 (3)	C14—C15—C19—F2	−172.7 (8)
C9—C10—C11—C12	0.8 (5)	C16—C15—C19—F1'	−70.5 (9)
C9—C10—C11—C13	179.5 (3)	C14—C15—C19—F1'	108.8 (8)
N2—N1—C12—O1	−174.0 (3)	C16—C15—C19—F3	128.5 (6)
C1—N1—C12—O1	5.0 (5)	C14—C15—C19—F3	−52.2 (6)
N2—N1—C12—C11	5.2 (5)	C16—C15—C19—F2'	36.4 (7)
C1—N1—C12—C11	−175.9 (3)	C14—C15—C19—F2'	−144.3 (6)
C10—C11—C12—O1	175.2 (3)	C16—C15—C19—F1	−121.3 (7)
C13—C11—C12—O1	−3.6 (5)	C14—C15—C19—F1	58.0 (7)

## References

[bb1] Bruker (1999). *SMART* and *SAINT* Bruker AXS Inc., Madison, Wisconsin, USA.

[bb2] Heinisch, G. & Kopelent, H. (1992). *Prog. Med. Chem.* **29**, 141–183. 10.1016/s0079-6468(08)70007-9 1475369

[bb3] Kolar, P. & Tisler, M. (1999). *Adv. Heterocycl. Chem.* **75**, 167–241.

[bb4] Sheldrick, G. M. (1996). *SADABS* University of Göttingen, Germany.

[bb5] Sheldrick, G. M. (2008). *Acta Cryst.* A**64**, 112–122. 10.1107/S0108767307043930 18156677

